# RNA-Based Analysis Reveals High Diversity of Plant-Associated Active Fungi in the Atmosphere

**DOI:** 10.3389/fmicb.2021.683266

**Published:** 2021-08-31

**Authors:** Yan Chen, Xishen Zhu, Ziqiong Hou, Yi Wang, Yunying Zhou, Ling Wang, Lin Liu, Jingrong Duan, Sauban Musa Jibril, Chengyun Li

**Affiliations:** State Key Laboratory for Conservation and Utilization of Bio-Resources in Yunnan, Yunnan Agricultural University, Kunming, China

**Keywords:** transcriptional activity, fungal community, plant pathogens, rRNA/rDNA, amplicon, atmosphere

## Abstract

Fungi are ubiquitous in nature; that is, they are present everywhere on the planet; understanding the active state and functional capacity of airborne microbes associated with health of human, animal, and plant is critical for biosafety management. Here, we firstly and directly proved that there were about 40% active fungi in the air *via* rRNA amplicon sequencing and imaging flow cytometry simultaneously. Amplicon sequencing analysis showed differences between structures of active and total fungal community; Ascomycota were dominant in the active community, while Basidiomycota have low transcriptional activity across all samples. Notably, plant pathogenic fungi were predominant in the air, and more than 50% were active, including not only several common plant pathogens but also biotrophic fungi (*Erysiphe* sp. and *Microbotryum* sp.) and host-specific pathogens, which were generally considered to be inactive after leaving the host. Putative plant pathogens of eight genera were found active across the sampling season, indicating their superior ability to obtain nutrients even in barren nutrient environments. Interestingly, we detected several potentially active unrecorded fungi in China (*Diatrype prominens*, *Septofusidium herbarum*, *Pseudomicrostroma glucosiphilum*, and *Uromycladium tepperianum*), which suggested that they spread over a long distance by air and may cause diseases under favorable conditions. Our results suggested that maintaining transmission in air is an essential feature of many fungi including plant pathogens regardless of being a biotrophic, hemibiotrophic, or necrotrophic group. Moreover, two potentially active human pathogens and one animal pathogen were captured, which indicated their potential risks. This study provided a new perspective for more comprehensive understanding of airborne fungi, including their multidimensional lifestyle, state, functioning, and potential pathogenic risk. It also laid the foundation for further prediction and management of airborne microbial communities, which would be of interest for public health and agriculture.

## Introduction

Airborne fungi play an important role in the global ecosystem (Woo et al., 2018), affecting not only precipitation, carbon, and nitrogen cycles (Pöschl et al., 2010; Haga et al., 2014; Hassett et al., 2015) but also the health of humans, plants, and animals (Brown and Hovmøller, 2002; Fisher et al., 2012; Golan and Pringle, 2017). The atmosphere is not the most extreme microbial habitat, and most of its features are consistent with other habitats (Womack et al., 2010). For many microbes, transport in the air is part of their life cycle (Brown and Hovmøller, 2002). In order to maintain their ability to survive in the atmosphere, they have adapted to the atmospheric environment and evolved survival strategies for long-distance dispersal and/or high-altitude diffusion (Imshenetsky et al., 1978; Griffin, 2004; Kellogg and Griffin, 2006; Joly et al., 2015). Many fungi including pathogenic fungi of plants and animals can spread over long distances or even intercontinental, causing, or exacerbating diseases (Brown and Hovmøller, 2002; Fisher et al., 2012; Golan and Pringle, 2017).

It has been found that microbes in air are metabolically active (mainly indicated as bacteria currently), and the residence time in the atmosphere is long enough to reproduce for some microorganisms (Womack et al., 2010). The function of fungi in the atmosphere depends on the physiological state of cells. Metabolically active vegetative cells have greater potential to transform atmospheric compounds and ultimately alter atmospheric chemistry than dormant spores (Sussman and Douthit, 1973). The ice nucleation efficiency of fungal cells may also be determined by their physiological state. Potentially active nutrient cells can form ice nucleation more effectively than fungal spores (Iannone et al., 2011; Després et al., 2012; Morris et al., 2013). Metabolically active fungi were isolated from the atmospheric samples, which could transform carboxylic acid, formaldehyde, and hydrogen peroxide compounds, which play a major role in the atmospheric chemical process (Ariya et al., 2002; Cote et al., 2008; Vaïtilingom et al., 2013).

In recent years, the application of molecular biology techniques including cloning approaches, qPCR, denaturing gradient gel electrophoresis (DGGE) (Fröhlich-Nowoisky et al., 2009; Li et al., 2010), and high-throughput sequencing are accelerating the research of atmospheric microbiome (Yamamoto et al., 2012, 2014; Woo et al., 2013; Cao et al., 2014; Dannemiller et al., 2014; Emerson et al., 2015; Womack et al., 2015; Banchi et al., 2018). Previous studies have shown that fungal spores and other biological particles account for a large proportion of aerosol particles in rain forests and in rural and urban environments. The microorganisms in the atmospheric are as diverse as in the terrestrial environments (Hughes et al., 2004; Eoin et al., 2007), and their community structure showed significant diurnal dynamics (Fierer et al., 2008) and seasonal cycles (Fröhlich-Nowoisky et al., 2009; Du et al., 2018).

Nevertheless, most of culture-independent studies of microbial community diversity only rely on sequence analysis of rDNA, which provides the overall information of living, dead, and dormant cells in a community. However, there may be fundamental differences in structure and composition between active and total communities. For example, it is the rare members of the total community that are dominant in the active community in many environmental systems (Campbell et al., 2011; Klein et al., 2016); using rDNA data alone may lead to an underestimation of the functional importance of rare taxa (Campbell et al., 2011; Aanderud et al., 2015). Therefore, sequence analysis based on rRNA in ribosomes and other reliable and efficient technology such as imaging flow cytometry were necessary to uncover the composition of the active community, because ribosomes are more abundant in active than in dormant cells (Muttray and Mohn, 1988; Prosser, 2002; Lennon and Jones, 2011; Womack et al., 2015). Additionally, imaging flow cytometry is an innovative, high-throughput technology that can provide a clear picture of each microbe cell or particle, as well as the ability to identify dead or live cells by fluorescent staining (McFarlin et al., 2013; Verma et al., 2018). One of the common methods to investigate total and active communities is to measure the community composition of DNA and RNA in ribosomes (Chicharo and Chicharo, 2008; Blazewicz et al., 2013; Womack et al., 2015; Denef et al., 2016). This approach has been applied to study active microbial communities on soil and on decaying plant materials (Rajala et al., 2011; Baldrian et al., 2012; Barnard et al., 2013; Schostag et al., 2015), but there are few reports on the metabolic activity of airborne microbial communities.

Klein et al. (2016) analyzed the metabolic activity of bacteria community in the atmosphere, and they found that the composition of the putatively active bacterial community (assayed *via* rRNA) differed significantly from that of the total bacterial community (assayed *via* rDNA), and rare taxa in the total community were disproportionately active relative to abundant taxa. Amato et al. (2017) studied the active and total microbial community in cloud water, and they uncovered the potential active fungal species mainly composed of saprophytic fungi from terrestrial or aquatic origins known for aerial dispersion, such as Pleosporales, Magnaporthales, Xylariales, and Conioscyphales in Ascomycota; and Pucciniales, Hymenochaetales, and Sporidiobolales in Basidiomycota. Another research about active and total fungal communities in the atmosphere over the Amazon rainforest by Womack et al. (2015) revealed significant differences in the composition of the total and active fungal communities in the atmosphere; and compared with other environments, fungal communities in the atmosphere were most similar to communities found in tropical soils and leaf surfaces. These studies indicate that the combination of rRNA and rDNA sequence analysis not only reveals the diversity of microbial communities in the atmosphere more comprehensively but also can evaluate the species with potential metabolic activity. However, no research focused on both diversity and function of active fungal communities in the atmosphere.

To our best of knowledge, at present, there is no *in situ* evidence of fungal growth in the air, although fungi can grow in the absence of carbon sources (i.e., fungi could grow on silica gel media as well as in distilled water and liquid culture media without carbon had been added) (Wainwright, 1987); and *Aspergillus*, *Penicillium*, *Eupenicillium*, and *Thysanophora* presented with the fastest transformation rate of malonic acid, which is the most abundant in organic aerosols (Cote et al., 2008) based on the result of previous studies. Therefore, transcriptional activity is more accurate to describe the results based on RNA sequencing at present study, mainly for the following reasons: the cell’s total RNA pool is mainly composed of rRNA (82–90%) (Tissières et al., 1959; Neidhardt and Magasanik, 1960), rRNA is a fundamental constituent of all known microorganisms, most rRNA found in a cell is ribosome associated as an integral structural component of ribosomes (Lindahl, 1975; Nomura et al., 1984), and rRNA is a product of transcription (Blazewicz et al., 2013).

In this study, we used comparative rRNA/rDNA amplicon sequencing to study the composition and structure of potentially active and total fungal community in the atmosphere of Kunming city, Yunnan Province of China, an area that is a typical low-latitude plateau with good air quality. We asked the following questions about the fungal communities inhabiting the atmosphere of Kunming: (1) What is the composition of the potentially active and total fungal communities in the atmosphere? Are there differences? (2) What are the major functional groups of the potentially active and total fungal communities in the atmosphere? (3) What are the potential plant pathogenic fungi in the atmosphere? If there are any, what are their potential activities? We aimed to set up a basis, expand the scope of biodiversity and biogeography research, provide information for understanding of diversity and function of atmospheric fungal community, and provide a basis for further studies on the prediction and even management of airborne microbial communities that would be of interest for public health and agriculture.

## Materials and Methods

### Site Description and Air Sampling

The study site is located within Yunnan Agricultural University, Northern Kunming, China. Air microbial samples were collected from the roof top of the Crop Protection Building (25°7′53.45″N, 102°44′55.36″E, altitude of 1,931 m), about 30 m above the ground; the sampling area is without interference from surroundings, and there are no major pollution sources nearby (see air quality data in Supplementary Table 1). Samples were collected using six independently modified air samplers (KC8704 air sampler, Changzhou Jinfen Instrument Co., Ltd., Jiangsu, China) Ambient air was drawn at an average flow rate of 12.5 L/min; samples were continuously collected from January 1, to July 31, 2018. The filter was changed each week; the filter holder was cleaned with 75% ethanol; and all the tools used for changing new filters, as well as new filters, were packaged separately in aluminum foil, then autoclaved at 121°C, and dried at 55°C previously to avoid contamination. After sampling, the filters were kept in a 50-ml sterile centrifuge tube, 10-ml RNA Later^TM^ (Beyotime, Shanghai, China) in each tube; stored at 4°C for 24 h; and then transferred to be stored at -20°C until downstream analyses.

### Nucleic Acid Isolation and cDNA Synthesis

Samples collected of each month were bisected for DNA and RNA extraction. DNA was extracted using QIAGEN DNeasy PowerSoil Kit (Qiagen Inc., Hilden, Germany) according to the manufacturer’s instructions. Soil RNA Mini Kit from Omega Bio-Tek (Guangzhu, China) was used for RNA extraction; then cDNA synthesis was performed using 5× all-in-one RT MasterMix (with AccuRT Genomic DNA Removal Kit) from Applied Biological Materials Inc. (Richmond, BC, Canada). All the steps mentioned above were carried out in a clean bench, and all centrifuge tubes and pipette tips were nucleic free. All RNA extraction steps were performed on ice and centrifuged at 4°C. The extracted DNA and samples were stored at -80°C until further use.

### Library Preparation and Sequencing

The V4 region of the 18S rRNA genes and transcripts (rRNA which was converted to cDNA) were amplified using the forward primer 528F (5′-barcode-GCGGTAATTCCAGCTCCAA-3′) and reverse primer 706R (5′-barcode-AATCCRAGAATTTCACCTCT-3′). The PCRs were carried out in 50-μl volume reactions with 25 μl 2^∗^taq PCR mix, 1 μl of Primer F (10 μM), 1 μl of Primer R (10 μM), 4 μl of dNTPs, 17 μl of ddH_2_O, and 2 μl of template DNA. Thermal cycling consisted of initial denaturation at 98°C for 10 min, followed by 30 cycles of denaturation at 95°C for 1 min, annealing at 55°C for 30 s, and elongation at 72°C for 30 s, and finally at 72°C for 5 min. The concentration and purity of DNA and cDNA samples were tested by 1% agarose gel electrophoresis, and samples were diluted with sterile water to 1 ng/μl. Multiplexed sequencing libraries were generated using Ion Plus Fragment Library Kit 48 rxns (Thermo Fisher Scientific, Waltham, MA, United States) following manufacturer’s recommendations. The library quality was assessed on the Qubit@2.0 Fluorometer (Thermo Fisher Scientific) and then sequenced on an Ion S5TMXL Ion at Novogene Co. Ltd. (Beijing, China).

### Sequence Processing and Data Statistical Analyses

The offline single-end reads were filtered according to the Cutadapt (V1.9.1) quality-controlled process (Martin, 2011) to obtain raw reads; then raw reads were assigned to samples based on their unique barcode and truncated by cutting off barcode and primer sequence to obtain raw reads. Quality filtering of the raw reads was performed using to QIIME (V1.9.1) (Caporaso et al., 2010) with the following options: -*q* ≤ 19, -*r* = 3, and -*p* < 0.75. The reads were compared with the reference database (Silva database) (Quast et al., 2013) using UCHIME algorithm (Edgar et al., 2011) to detect and remove chimera sequences to obtain clean reads. The operational taxonomic units (OTUs) with a similarity threshold of 97% were selected with the Uparse software package (v7.0.1001) (Edgar, 2013), singletons were filtered before OTU clustering for improving calculation efficiency, while all clean read sequences (including singleton) were aligned with the representative sequence (the sequence with the highest occurrence frequency), and any matched sequences were retained to calculate the abundance of OTUs. The representative sequence for each OTU was selected for annotation using the Silva Database (SSU rRNA) (Quast et al., 2013).

α-Diversity indices [Observed Species, Chao1, Shannon, Simpson, abundance-based coverage estimator (ACE), good coverage, and phylogenetic diversity (PD) whole tree] and beta diversity on Bray–Curtis and unweighted_unifrac were calculated with QIIME (Version 1.7.0). The dilution curve, rank abundance curve, species accumulation curve, non-metric multidimensional scaling (NMDS), and hierarchical clustering tree based on UPGMA by unweighted_unifrac distances were performed by Vegan and ggplot2 package in R software (Version 2.15.3). Paired *t*-test of α-diversity indices were conducted by SPSS 16.0; the average value and standard deviation were used to represent each index in the results; both Bray–Curtis and unweighted_unifrac by *t*-test were used for analysis of β-diversity differences between active and total fungal communities. Analysis of similarities (Anosim), multiresponse permutation procedure (MRPP) based on Bray–Curtis, and permutational multivariate analysis of variance (Adonis) on Bray–Curtis were performed using Vegan package in R software (Version 2.15.3); and analysis of molecular variance (Amova) based on unweighted_unifrac was performed in Mothur (Version 1.33.3) (Schloss et al., 2009) to determine whether there were significant differences between active and total fungal communities. Spearman’s correlation was used to test the correlation between relative abundance and rRNA:rDNA ratio. *p* < 0.05 indicates significant correlation.

### Ecological Function Assignment and Temporal Dynamics Analysis of Airborne Fungi

To obtain putative ecological functions from fungal OTUs at the genus or species level, the fungal OTU table was submitted to FUNGuild website^1^ (Nguyen et al., 2016) to do ecological function predication. For a more detailed assignment, we also reviewed relevant literatures and allocated known fungal into functional categories. The species distribution status in the world was queried on GBIF (https://www.gbif.org; March 21–30, 2019). Data of temperature, relative humidity, wind speed, and precipitation were recorded according to the reports of Chinese National Meteorological Center.^2^ The air quality data come from a local website.^3^

Core genera were defined as the genus occurring in all samples in the annotation table. All OTUs that had been annotated to species level in the annotation table previously were identified by BLAST matches in National Center for Biotechnology Information (NCBI); the species with the percentage of identification greater than 98% as the putative species (and species with percentage of identification <98% were removed; the alignment result with percentage of identification = 100%, but could not be identified to specific species were marked as “sp.”) were selected for functional group division and subsequent analysis based on the latest sequence BLAST in NCBI. The rRNA:rDNA ratio of each was calculated to analyze the temporal dynamics of transcriptional activity.

### Air Sampling and Detected by FlowSight^®^ Imaging Flow Cytometry

Samples of PM_2.5_ (air fine particulate matter, particulate matter with aerodynamic diameter below 2.5 μm), PM_10_ (air coarse particulate matter, particulate matter with aerodynamic diameter below 10 μm), and total suspended particulate (TSP) were collected from the same site as 18S V4 amplicon sequencing samples from April 1 to 3 conducted by three high volume air samplers (Thermo Electron Corp., Waltham, MA, United States): one was equipped with PM_2.5_ fractionating inlet, another one was equipped with a PM_10_ fractionating inlet, and the third one was without fractionating inlet. All samples were continuously collected for 60 h with an average flow rate of 1.13 m^3^/min, resulting in approximately 4,068 m^3^ of air flow-through per sample. Particulate matter was collected on 20.32 × 25.4 cm^2^ Tissuquartz filters (PALL, Port Washington, NY, United States), which was autoclaved at 121°C and dried at 55°C previously. A piece of 1/48 of the filter from each of the samples was cut and placed in 1.5-ml centrifuge tubes filled with sterilized 1× phosphate-buffered saline (PBS), static for 40 min, then centrifuged at 1,000 r/min for 20 min, sucked out precipitation, gentle vortex, and stained using propidium iodide (PI) according to the manufacturer’s instructions (Yeasen Co., Ltd, Shanghai, China). Image acquisition was carried out using a 20×objective on a FlowSight^®^ Imaging Flow Cytometer (Amnis^®^, Luminex, Chicago, United States) equipped with a 488-nm laser. A total of 20,000 single, in-focus images were captured on FlowSight^®^ per sample. PI-stained fluorescence and chlorophyll spontaneous fluorescence were also detected.

The IDEAS^®^ software (Version 6.2) was used for image analysis and cell counting. At first, the best-focused images were selected from raw images; then cells of best-focused images were gated according to “Area” (0–500 μm^2^) and “Aapect Ratio” (0–1) into four gates. Cells were further selected with parameter “Circularity” from 1 to 15 (eliminate debris of plants/animals and fiber, etc.) and “Length” from 0 to 120 μm from the four groups and then classified according to parameter of length and result of fluorescence: the cells with length of ≤5 and ≤20 μm (considered as fungi), the cells with length <5 μm (considered as bacterial), the length of most protozoa is larger than 20 μm; cells/particles successfully stained with PI were considered as dead cells, and chlorophyll auto-fluorescence cells/particles were considered as green plants or algae; the number of each gate was counted for data analyses.

## Results

### Sequencing Summary

A total of 540,388 and 532,079 qualified reads with average lengths ranging from 304 to 310 nucleotides for 18S rDNA and rRNA sequencing were acquired, respectively, with an average of 76,605 ± 8,786 reads in each sample (Table 1 and Supplementary Table 3). In total, 1,994 OTUs were obtained by clustering at 97% sequence similarities, including 686 OTUs in rDNA sequencing subset and 1,144 OTUs in rRNA sequencing subset, and 43.1% of the total OTUs (852 OTUs) were common in both subsets. The number of unique OTUs that occurred in rDNA sequencing subset and RNA sequencing subset was 834 and 292, respectively; the proportion of unique OTUs out of total OTUs was 42.2 and 14.8%, respectively. There were 628 OTUs that were assigned to fungi, 48 OTUs belong to metazoans (Animalia), and the remaining 1,247 OTUs were indicated as eukaryotic (Apicomplexa, Chlorophyta, Diatomea, Streptophyta, and other unidentified_Eukaryota) in Silva database. There were 77.7% fungal OTUs (488 OTUs) common in both rDNA and rRNA sequencing subsets, and the unique OTUs in rDNA and rRNA sequencing subset were 89 and 51, accounting for 14.2 and 8.1%, respectively. All of the following data analyses were based on OTUs that were annotated to fungi.

**TABLE 1:** Sequence summary statistics based on 18S rDNA and rRNA metagenomics.

Items	Total metagenomics result	Fungal community
Raw sequences	1,139,258	–
Filtered sequences	1,072,467	–
Mean filtered sequences/sample	76,605 ± 8,786	–
Number of OTUs	1,994	628
Mean OTUs/rDNA libraries	733 ± 155.5	264.4 ± 95.1
Mean OTUs/rRNA libraries	499 ± 111.7	254.4 ± 70.6
OTUs shared across all libraries	75	54
rDNA OTUs	1,686	572
rRNA OTUs	1,144	539
OTUs shared between rDNA and rRNA libraries	852 (43.1%)	488 (77.7%)
OTUs in rDNA but not rRNA libraries	834 (42.2%)	89 (14.2%)
OTUs in rRNA but not rDNA libraries (phantoms)	292 (14.8%)	51 (8.1%)

*OTUs, operational taxonomic units.*

### The Composition and Structure of Active Fungal Community Were Significantly Different From Those of Total Community

All of the α-diversity indices such as Richness, Chao1, ACE, Shannon, Simpson, good coverage, and PD whole tree (see Supplementary Figure 4 for hierarchical clustering tree) were not significantly different between total fungal community and the potential active fungal community based on result of *t*-test (Supplementary Table 4). Both comparisons of the β-diversity based on Bray–Curtis distance and unweighted_unifrac calculation showed that there were no significant difference between two communities with *p* = 0.306 (on Bray–Curtis) and *p* = 0.284 (on unweighted_unifrac).

**FIGURE 1:**
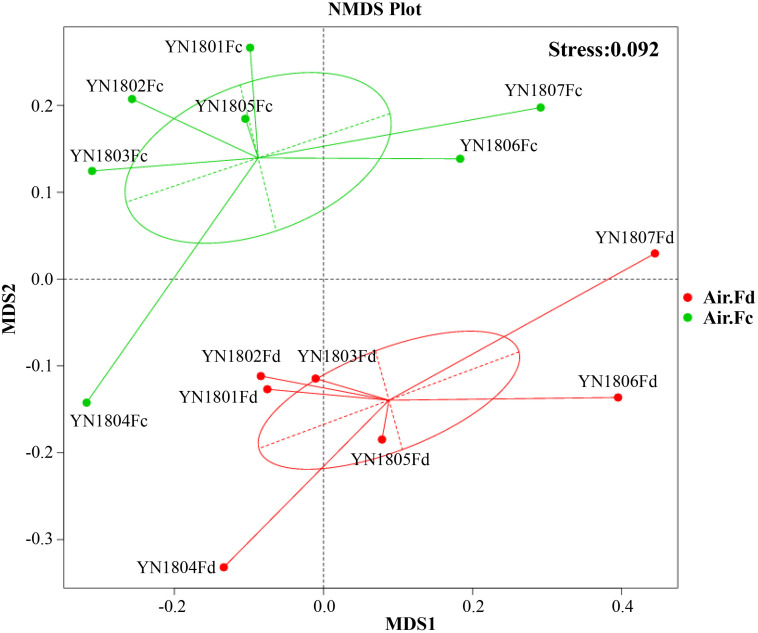
Bray–Curtis matrices visualized using non-metric multidimensional scaling (NMDS) analysis showing distribution of rDNA (red dots, Air.Fd) and rRNA (green dots, Air.Fc) sequencing samples of fungal communities in the atmosphere.

The NMDS showed distinct clustering with regard to two communities in Figure 1. Algorithm analysis results to test the differences of community structure between groups showed significant differences between total and potentially active fungal communities (Anosim, *R* = 0.392, *p* = 0.008; MRPP, *A* = 0.083, *p* = 0.06; Adonis, *R*^2^ = 0.209 (0.791), *p* = 0.007; Amova, *F*s = 3.162 and *p* = 0.004).

**FIGURE 2:**
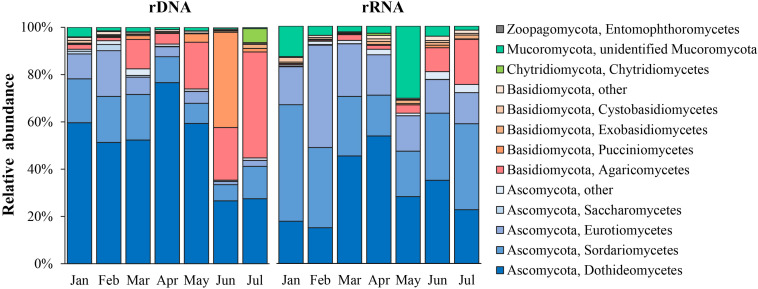
Class-level taxonomic composition of rRNA and rDNA fungal communities in the atmosphere.

A total of five phyla, 25 classes, 66 orders, 112 families, 137 genera, and 154 species were identified for the common fungal OTUs in both total and active communities. In the total fungal community (rDNA sequencing subset), Ascomycota and Basidiomycota were dominant with relative abundance of 67.9 ± 23.2% and 29.7 ± 22.5%, respectively; a very low relative abundance was observed in Chytridiomycota (1.3 ± 2.1%), Mucoromycota (1.0 ± 0.7%), and Zoopagomycota (0.1%). In the active fungal community (rRNA sequencing subset), Ascomycota, Basidiomycota, and Mucoromycota dominated with relative abundance with 76.1 ± 12.4, 17.6 ± 13.7%, and 6.0 ± 5.0%, respectively. As shown in Figure 2, the common dominant classes in both the total fungal community and active community were Dothideomycetes (mean relative abundance = 50.5 ± 16.7% in rDNA sequencing subset; 33.5 ± 11.8% in rRNA sequencing subset), Sordariomycetes (13.9 ± 4.9%; 32.5 ± 18.1%), and Eurotiomycetes (7.2 ± 5.7%; 22.2 ± 9.2%) in Ascomycota. In the phylum Basidiomycota, the composition of dominant classes was obviously different in total community and active ones; Agaricomycetes (15.3 ± 14.3%) and Pucciniomycetes (6.9 ± 13.7%) were dominant in total community, while in the active community, only Agaricomycetes dominated with relative abundance of 3.5 ± 3.1%. The average relative abundance of Mucoromycota (mainly was annotated to Mucorales) only accounted for 1% in total community, while it was dominant in the active community with average relative abundance accounting for 10.5 ± 9.9%.

The 10 most dominant orders in total community were Capnodiales (23.1 ± 14.0%) and Pleosporales (19.8 ± 6.4%) of Dothideomycetes; Xylariales (5.9 ± 2.6%) and Hypocreales (2.4 ± 1.5%) of Sordariomycetes; Helotiales (2.8 ± 2.2%) of Leotiomycetes and Chaetothyriales (2.8 ± 2.2%) of Eurotiomycetes, which were in Ascomycota; Agaricales (7.8 ± 8.4%), Polyporales (6.2 ± 4.7%), and Corticiales (2.8 ± 3.6%) of Agaricomycetes; Pucciniales (3.7 ± 6.9%) of Basidiomycota. In the active community, the 10 most dominant orders were Pleosporales (18.1 ± 10.2%), Xylariales (12.8 ± 4.0%), Eurotiales (8.8 ± 5.4%), Capnodiales (7.1 ± 5.9%), Sordariales (7.2 ± 5.0%), Chaetothyriales (3.4 ± 2.2%), Helotiales (3.6 ± 2.5%), Hypocreales (2.8 ± 1.6%), Agaricales (9.0 ± 10.7%), and Mucorales (4.9 ± 6.4%).

The top 10 most abundant genera in total community included *Cladosporium* (5.5 ± 2.4%), *Boeremia* (2.9 ± 2.3%), *Alternaria* (1.5 ± 1.1%), *Arthrinium* (1.1 ± 0.5%), unidentified_Pleosporales (1.0 ± 0.7%), and *Sclerotinia* (0.9 ± 0.6%) in Ascomycetes; *Psathyrella* (2.9 ± 4.3%), *Gymnosporangium* (2.5 ± 5.7%), and *Trametes* (1.3 ± 1.3%) in Basidiomycota; and *Synchytrium* (0.8 ± 2.0%) in Chytridiomycota. The top 10 most abundant genera of the potentially active community included eight genera in Ascomycota, i.e., *Arthrinium* (4.6 ± 1.7%), *Aspergillus* (2.9 ± 1.8%), *Alternaria* (2.8 ± 1.8%), *Cladosporium* (2.8 ± 2.4%), *Sclerotinia* (2.0 ± 1.7%), *Boeremia* (1.7 ± 0.8%), *Penicillium* (1.0 ± 0.7%) and unidentified_Pleosporales (0.9 ± 0.8%), one genus in Basidiomycota (*Psathyrella*, 3.7 ± 4.9%), and *Rhizopus* (2.3 ± 3.2%) in Mucoromycota.

The community composition varied obviously in different sampling months for both the total and active communities (Figure 2). In the total fungal community, Ascomycota was predominant from January to April, with an average relative abundance of 86.3%; Basidiomycota has the relative abundance of 11.7%; from May to July, the proportion of Basidiomycota increased gradually and becomes higher than that of Ascomycota (the average relative abundance of Basidiomycota was 53.6%, and that of Ascomycota was 43.5%). In the potentially active fungal community, Ascomycota was predominant across sampling months, average relative abundance was 76% (50.9–92.5%); and the relative abundance of Basidiomycota (6–48.4%, on average of 17.6%) and Mucoromycota (0.6–20%, on average of 5%) was lower.

### Putative Species and Their Ecological Functions of Potentially Active Fungi

**FIGURE 3:**
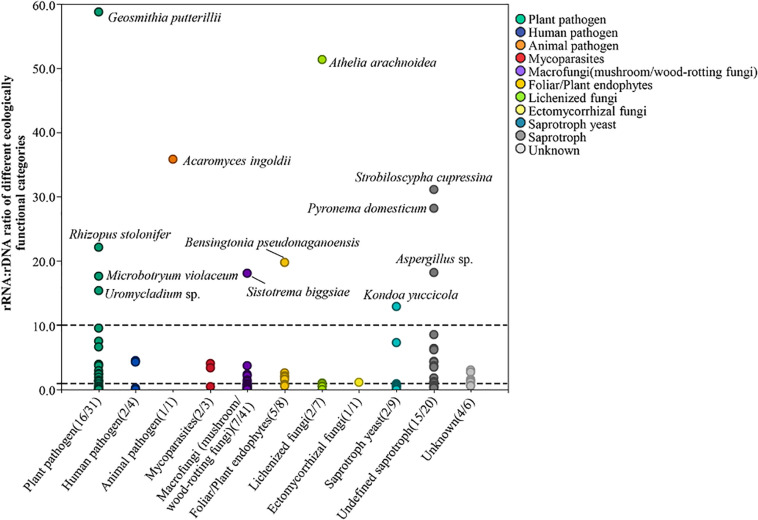
Potential transcriptionally active fungi of different ecologically functional categories. The numbers in brackets in each category indicate the number of transcriptionally active fungal species of the group and the total number of the group, respectively.

By combining the results of functional prediction *via* FUNGuild and description of relevant literatures, 131 fungi, which were further species identified by BLAST matches in NCBI, were grouped into 10 ecological categories (plant pathogen, human pathogen, animal pathogen, mycoparasites, macrofungi (mushroom/wood-rotting fungi), foliar/plant endophytes, lichenized fungi, ectomycorrhizal fungi, saprotroph yeast, and undefined saprotroph) and an additional group of unknown. The species number of transcriptionally active fungi and total number of each category are presented in brackets in Figure 3; the total species number of each category was 31, 4, 1, 3, 41, 8, 7, 1, 9, 20, and 6. In total, 57 putative fungi with potential transcriptional activity (i.e., rRNA:rDNA ratio > 1) were detected (Supplementary Table 6) including 16 plant pathogens, 16 fungi of saprotroph, two human pathogens, one animal pathogen, five foliar/plant endophytes, seven fungi of macrofungi(mushroom/wood-rotting fungi), two lichenized fungi, one ectomycorrhizal fungi, and one mycoparasites. The proportion of fungi with transcriptional activity varied in different ecological functional categories, i.e., plant pathogen (51.6%), human pathogen (50%), mycoparasites (66.7%), macrofungi (mushroom/wood-rotting fungi, 17.1%), foliar/plant endophytes (62.5%), lichenized fungi (28.5%), ectomycorrhizal fungi (100%), saprotroph yeast (22.2%), and undefined saprotroph (75.0%).

**FIGURE 4:**
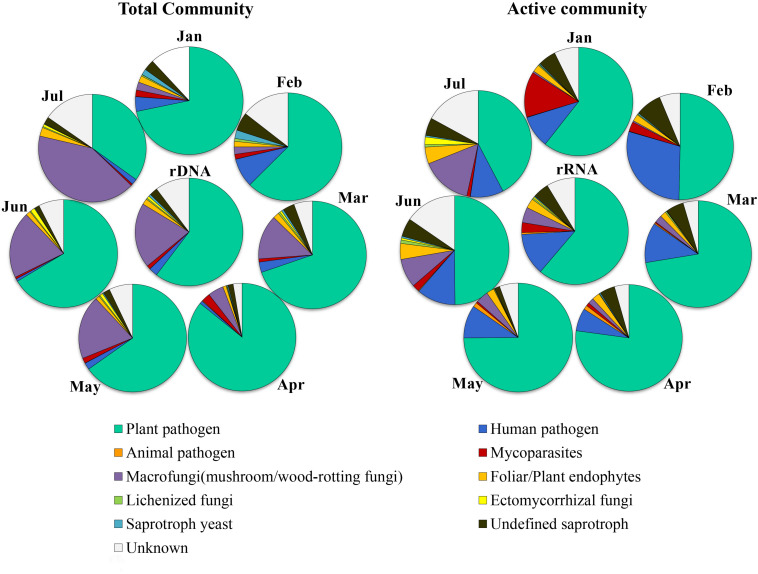
Dynamics of relative abundance of major ecologically functional categories in different months in potential active fungal community (rRNA) and total fungal community (rDNA).

The relative abundance of each ecological category in total and active fungal communities in different months is shown in Figure 4, among which plant pathogen dominated in both total and potentially active fungal communities with average proportions of 65.2% (34.9–85.8%) and 61.1% (42.3–77.2%), respectively. The relative abundance of macrofungi in the total community ranged from 2.4 to 41.5%, which was lower from January to April and then increased significantly from May to July. In addition, the variation trend of the abundance in the potentially active community was the same as that in total community, but with lower proportion (0.5–15.5%). The relative abundance of other categories in total community was very low except for the unknown group, i.e., human pathogen (0.8–8.7%), animal pathogen (0–0.2%), mycoparasites (0.5–2.6%), foliar/plant endophytes (1–2.6%), lichenized fungi (0.2–0.9%), ectomycorrhizal fungi (0–1.3%), saprotroph yeast (0.2–2.5%), undefined saprotroph (1.4–5.5%), and unknown (2.7–15.3%). However, in the potentially active community, the abundance of human pathogen in all months was much higher (6.9–29.1%), and the proportion of mycoparasites was much higher in January (13.6%); the abundance of saprotroph (including saprotroph yeast and undefined saprotroph) changed little in different months (ranged from 1.5 to 8.3%).

### Temporal Dynamics of Transcriptional Activity of Airborne Fungi

There were 55 core OTUs (i.e., OTUs shared among all the samples) in the present study, and of them, 27 OTUs were annotated at genus level based on annotation result (Table 2). Exceptionally, eight genera with transcriptional activity were detected across all the sampling months, including *Arthrinium*, *Aspergillus*, *Erysiphe*, *Knufia*, *Microascus*, *Penicillium*, *Torula*, and *Rhizopus* with the rRNA:rDNA ratio ranging from 1.07 to 67. Their transcriptional activity changed dynamically from month to month, and rRNA:rDNA ratios of most genera were the highest in April. In addition, the transcriptional activity of other fungi, such as *Boeremia*, *Cladosporium*, *Pestalotiopsis*, *Sclerotinia*, *Geastrum*, and *Lactarius*, also showed the highest transcriptional activity from March to May.

**TABLE 2:** Temporal dynamics of the potential active of 23 core airborne fungal genera.

Genus	rRNA:rDNA ratio						
	Jan	Feb	Mar	Apr	May	Jun	Jul
**Ascomycota**							
*Alternaria*	0.71	**1.31**	**15.08**	**10.02**	**1.32**	**1.26**	0.65
*Arthrinium*	**3.90**	**4.46**	**4.72**	**12.16**	**6.37**	**3.39**	**1.59**
*Aspergillus*	**2.83**	**5.85**	**15.79**	**36.44**	**7.11**	**9.66**	**3.96**
*Boeremia*	0.81	**1.18**	**1.31**	**4.38**	0.51	0.41	0.31
*Bradymyces*	0.91	**1.32**	**7.50**	**6.50**	**1.90**	**2.35**	**3.84**
*Cladosporium*	0.21	0.31	0.87	**2.04**	0.28	0.33	0.29
*Diatrype*	**6.00**	0.93	**7.10**	**2.00**	0.94	**1.75**	0.96
*Erysiphe*	**1.45**	**2.40**	**4.91**	**9.83**	**1.91**	**3.07**	**1.95**
*Fusarium*	0.69	0.29	**2.58**	**2.67**	0.60	**2.96**	**2.08**
*Knufia*	**5.32**	**2.48**	**12.33**	**13.57**	**1.07**	**1.46**	**1.23**
*Microascus*	**2.45**	**6.73**	**22.50**	**32.50**	**3.00**	**4.80**	**2.00**
*Neurospora*	**10.11**	**3.04**	**1.71**	**1.03**	0.33	0.92	**1.28**
*Penicillium*	**2.55**	**8.13**	**25.88**	**67.00**	**10.35**	**9.90**	**3.65**
*Pestalotiopsis*	**1.11**	0.43	**6.80**	**5.35**	0.96	**1.06**	**1.11**
*Plectosphaerella*	0.07	**2.08**	**2.50**	**1.67**	0.62	0.23	0.16
*Sclerotinia*	0.46	**1.40**	**4.05**	**4.31**	**2.67**	**3.18**	**1.52**
*Torula*	**1.09**	**1.67**	**3.25**	**3.42**	**2.67**	**2.75**	**1.88**
**Basidiomycota**							
*Dioszegia*	0.53	**1.17**	**3.00**	0.83	0.22	0.50	**1.50**
*Geastrum*	0.40	0.33	**2.00**	**6.01**	**1.07**	0.75	0.46
*Lactarius*	**2.76**	**2.54**	**2.83**	**5.50**	**1.39**	**1.48**	0.93
*Psathyrella*	**6.44**	**3.22**	**1.75**	**4.52**	0.95	**1.29**	**1.14**
*Rhizoctonia*	0.75	**4.00**	**1.38**	0.67	0.20	0.45	0.28
*Trametes*	0.15	0.29	0.40	**1.41**	0.21	0.26	0.36
**Mucoromycota**							
*Rhizopus*	**4.28**	**5.17**	**5.31**	**8.78**	**38.32**	**6.25**	**2.30**

*Bold numbers in the table indicate the rRNA:rDNA ratio of the genus was higher than 1 in sampling months.*

### Potentially Active Fungi Detected by FlowSight^®^ Imaging Flow Cytometry

**FIGURE 5:**
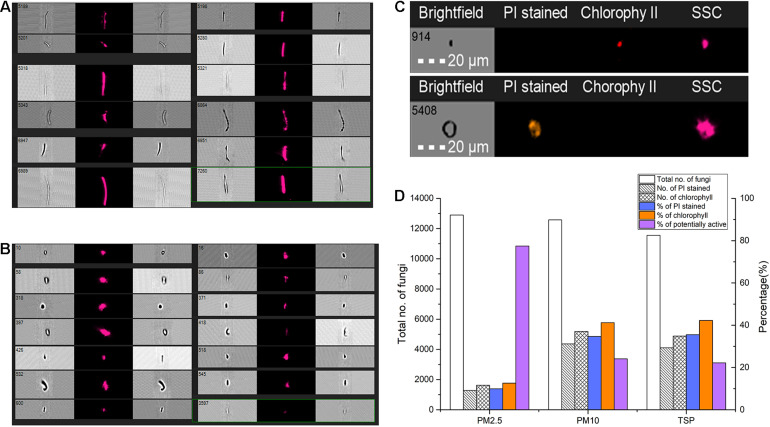
Potential transcriptional active fungi detected by FlowSight^®^ imaging flow cytometry. **(A,B)** Different forms of particles in FlowSight Amnis’s working process. **(C)** The imaging of different particles or fungal spores with propidium iodide (PI) staining. **(D)** The proportion of potentially active fungi in samples of different aerodynamic diameters.

The FlowSight^®^ imaging flow cytometry results of air microbial samples with different aerodynamic diameters showed that in the gate of diameter between 5 and 20 μm (considered as fungi), the proportion of dead cells (i.e., cells/particles successfully stained by PI staining) in samples of PM_2.5_, PM_10_, and TSP was 9.94, 34.71, and 35.54%, respectively, and the proportion of cells with chlorophyll spontaneous fluorescence was 12.60, 41.20, and 42.26%, respectively. Therefore, the proportion of potentially active fungi in samples of PM_2.5_, PM_10_, and TSP was 77.46, 24.09, and 22.20%, respectively, and the average was 41.25% (Figure 5).

## Discussion

### Robust Evidences of Fungal Activity in the Air

Microorganisms are critical to the functioning of terrestrial and aquatic ecosystems; they play a role in the functioning of the atmosphere as well. Due to recent advances in molecular techniques and DNA sequencing capabilities, more and more reports emerged on global microbiomes through cultivation-independent molecular approach (Bahram et al., 2018; Delgado-Baquerizo et al., 2018; Nayfach et al., 2020). However, studies of the global microbiome are not evenly distributed, and little is known about the biology of the atmosphere relative to aquatic and terrestrial habitats.

In recent years, several studies with short-term and local sampling on diversity of atmospheric microbial *via* metagenomics have been reported, mainly focused on Europe (Fröhlich-Nowoisky et al., 2009; Nicolaisen et al., 2017; Banchi et al., 2018), the United States (Yamamoto et al., 2012), South Korea (An et al., 2017; Woo et al., 2018), Canada (Chen et al., 2018), Northern China (Cao et al., 2014; Yan et al., 2016; Wei et al., 2019), etc. However, there are few reports on atmospheric microbiome in the low-latitude plateau of China.

This is the first study to examine active fungal community in the atmosphere using RNA-based approach and imaging flow cytometry. Both results consistently indicated that more than 40% of airborne fungi have potential transcriptional activity, which is much higher than that in our previous perception. There is robust evidence to directly prove that there are potentially active fungi in the air. Bowsher et al. (2019) demonstrated that 16S rRNA/rRNA gene ratios and cell activity staining reveal consistent patterns of microbial activity in plant-associated soil. They also indicated the 16S ratio and CTC methods report comparable patterns of activity that can be applied to observe ecological dynamics over time, space, or experimental treatment. It is of interest to distinguish active and inactive microbial cells and taxa to understand their functional contributions to ecosystem processes and to understand shifts in microbial activity in response to change. Also, it can contribute to exploring whether the active fungal group can survive and even reproduce in the atmosphere using barren substrates. Is the atmosphere for them, as same as soil and water? Do they play a broader ecological role among different spheres of the Earth? Importantly, the first step in further research is needed to understand if this transcriptional activity equals really metabolic activity or if it is related to spores.

### The Structure Between Active and Total Fungal Community Was Significantly Different

In the present study, we firstly conducted long-term sampling analysis in a typical low-latitude plateau region of the northern hemisphere; the result showed that Ascomycota and Basidiomycota were dominant in total fungal community in the atmosphere, with the relative abundance of 30.6–89.9% and 8.2–62.6%, respectively, which is consistent with most previously reported results in the world. On the other hand, a few studies reported Basidiomycota as predominant, rather than Ascomycota (Fröhlich-Nowoisky et al., 2009; Womack et al., 2015). Different results may be related to sampling season, local environment, sequencing method, and primers used.

The structure between active and total fungal community was significantly different; Ascomycota was predominant with the average relative abundance of 76%, while that of Basidiomycota was only 17.6%. Dothideomycetes, Sordariomycetes, and Eurotiomycetes in Ascomycota and Agaricomycetes in Basidiomycota were dominant in the active community, which was consistent with the result of only previous report of active fungal community in the atmosphere over the Amazon rainforest canopy (Womack et al., 2015). If these results can be confirmed, Ascomycota predominant in the atmosphere should be a general rule. Ascomycota usually have single-celled or filamentous vegetative growth forms that are small enough to become aerosolized, whereas many of the Basidiomycota are too large to be easily aerosolized other than in the form of metabolically inactive spores (Womack et al., 2015). The result in present study also indicated Ascomycota and Basidiomycota have different adaptability to the atmosphere. Eight orders including Pleosporales, Capnodiales, Chaetothyriales, Eurotiales, Helotiales, Xylariales, and Hypocreales in Ascomycota; one order (Agaricales) in Basidiomycota; and one order (Mucorales) in Mucoromycota were the top 10 dominant orders. Among which Pleosporales and Xylariales have been reported to be abundant in the active communities in cloud water by Amato et al. (2017).

Furthermore, significant differences in community structure between total and potentially active fungal communities were also in accordance with the previous studies by Baldrian et al. (2012); Womack et al. (2015), and Klein et al. (2016). This emphasizes the importance of diversity and structure of the active microbiome in future research; deeply understanding both structure and function of microbial communities can help to understand their role in the atmosphere and other environment. Previous studies reported significant diurnal dynamics of atmospheric fungal communities (Fierer et al., 2008) and seasonal cycles (Fröhlich-Nowoisky et al., 2009; Du et al., 2018). In the present study, the community composition varied obviously in different sampling months for both the total and potentially active community. Basidiomycota increased significantly with the onset of the rainy season, which is consistent with the results of Nicolaisen et al. (2017); abundances of Basidiomycota were significantly higher in autumn than in early spring. This may be due to rainfall, which promotes growth, spore production, and diffusion of Basidiomycetes fungi, especially mushrooms and wood-rotting fungi (Chen et al., 2018). Our result indicated that the species number and relative abundance of Agaricomycetes were significantly increased with the increase of rainfall; i.e., compared with the average species number of January to April with that of May to July, it rapidly increased from 12.5 to 39.3, and the same trend was found for the average relative abundance, which increased from 0.36 to 9.31%, accordingly. In addition, the relative abundance of Pucciniomycetes and Exobasidiomycetes also increased significantly. Although the proportion of relative abundance of Basidiomycota (0.5–15.5%) was much lower in the potentially active community, the variation trend of the abundance was consistent with that in total community, which indicated that most of them may be dormant since the sampling period is not the abundant sporulation period of native macrofungi.

### Temporal Dynamics of Transcriptional Activity of Airborne Fungi

This happens to be the first time to describe the temporal dynamics of potential transcriptional activity of fungi in the air *via* molecular technique. We speculate that as temperature rises and rainfall increases, along with higher wind speed promoting more fresh spore release into the air from the ground, they may keep transcriptional activity. Additionally, most detected fungi were active in April, suggesting that seasonal variation of fungal activity is caused by the alternation from dry season to rainy season occurring in April (Supplementary Figure 5).

### Rare Taxa in the Airborne Fungal Community Were Disproportionately Active

Some rare taxa may be key species on network in some communities or play important ecological function. Recently, study on estimate of the sequenced proportion of the global prokaryotic genome, which was based on large-scale sequence alignment between Earth Microbiome Project (EMP), released data and the sequenced genomes in the public database, and the result highlighted that there were a large number of rare taxa that are not deeply explored (Zhang et al., 2020).

The results of the present work reveal significant negative correlation between the relative abundance of the fungi in total community and rRNA:rDNA ratio (*R*^2^ = 0.305, *p* < 0.001) indicates that some of potentially transcriptional active species could occur with very low relative abundance in the total community. In addition, by comparing the top 35 abundant putative species in the potentially active community with those of total community, it was found that only five putative species (i.e., *Arthrinium* sp., *Aspergillus* sp., *Rhizopus oryzae*, *Knufia petricola*, and *Penicillium* sp.) were in the top 20% abundant group of the active community, but the relative abundance of the remaining 30 species in the active community only ranged from 0.001 to 0.05%.

Both results indicate that rare taxa in the airborne fungal community were disproportionately active, a phenomenon also observed in the atmosphere (Klein et al., 2016), soil (Dlott et al., 2015), marine (Campbell et al., 2011), and wastewater anaerobic digester (Jia et al., 2019) for bacterial communities. There may be fundamental differences in the structure and composition of active and total communities. Using rDNA data alone is limited in determining the diversity and activity state of the microbial community, which may underestimate the potential transcriptional activity of the rare groups (Campbell et al., 2011; Aanderud et al., 2015). They may play an important role in the biosphere; however, little research has been done on them, and their important ecological functions remain undiscovered until now.

### Ecological Functions of Potentially Active Fungi

To the best of our knowledge, published research has rarely focused on the function of airborne microbes with traits particularly well-suited for survival and potential activities in extreme environments, although it has been proposed by Womack et al. (2015). We allocated 131 putative species of fungi into 10 ecologically functional categories referring to literatures and functional annotation result in FUNGuild, as well as activity ability evaluation based on rRNA:rDNA ratio for each category. The results indicated that different functional groups of fungi have different adaptability to the atmosphere: some groups may be able to survive in the air for long time, while others may only stay for a short time and settle to the ground very fast.

#### Transcriptionally Active Plant Pathogenic Fungi in the Air

Our results showed that plant pathogenic fungi are the dominant group in the air, which is consistent with the study on the community characteristics of PM_2.5_ atmospheric bacteria and fungi after biomass combustion disturbance in a rural area of the north China plain. The abundant species were *Alternaria* spp., *Bipolaris* spp., *Botrytis* spp., *Fusarium* spp., and other plant pathogens, which may survive in the form of hypha or spore (Wei et al., 2019). A study by Nicolaisen et al. (2017) reported *Botrytis cinerea*, *Sclerotinia sclerotiorum*, *Blumeria graminis*, *Microdochium nivale*, *Venturia* spp., *Pyrenophora teres*, *Pyrenophora bromii*, *Phoma* spp., *Claviceps purpurea*, *Phaeosphaeria* sp., *Tilletia walkeri*, etc., as the major plant pathogenic fungi in the atmosphere. Chen et al. (2018) in the Canadian agricultural experimental station showed that the genera of plant pathogenic fungi with high abundance were *Cladosporium*, *Alternaria*, *Fusarium*, *Pestalotiopsis*, *Ophiognomonia*, *Erysiphe*, *Entyloma*, *Ustilago*, etc., which also appeared in the present study.

There is no report focus on diversity of plant pathogenic fungi with potential transcriptional activity in the atmosphere previously. According to our result, about 50% of putative plant pathogens have potential transcriptional activity in the atmosphere. Firstly, the group of common plant pathogens has a wide host range, such as genera of *Alternaria*, *Fusarium*, and *Rhizopus*. The species most commonly associated with plant disease are, e.g., *Alternaria alternata*, which causes wheat black spot, sorghum grain mold, and black spot of soybean; *Alternaria solani* causes early blight on potato and tomato; *Fusarium graminearum* causes seedling blight on rice and maize, wheat scab, maize *Fusarium* ear rot, sorghum *Fusarium* stem rot, soybean root rot, etc., which not only cause loss of crop yield but also seriously reduce the quality of crops by producing toxins; *Rhizopus oryzae* can cause soft rot of *jackfruit*; *Rhizopus stolonifer* usually causes post-harvest disease of peach, grape, jujube, strawberry, etc. (Guo et al., 2015). rRNA:rDNA ratios of these genera were greater than 1, i.e., *Alternaria* (1.86), *Fusarium* (1.45), and *Rhizopus* (9.81), which indicated they have potential transcriptional activity; probably as a result of long-term adaptive evolution in the air, the atmosphere could be a reservoir for them.

Surprisingly, typical biotrophic airborne pathogens such as *Erysiphe* sp. have rRNA:rDNA ratio >1 across all sampling months, and probably the most important reason may be due to the wide distribution of host plant (including peas, broad beans, alfalfa, *Melilotus*, and *Astragalus*; Guo et al., 2015), which provided wide hyphae or spore sources. Our results showed that some biotrophic fungi can be detected in the air year-round, suggesting that these fungi have alternative ways of survival and dispersal in air for long time. Many fungi with long-distance wind dispersal patterns are pathogens on major crop disease outbreaks worldwide, such as *Puccinia* spores, which are spread by wind over long distances in the air and cause stem rust in North America (Eversmeyer and Kramer, 2000), wheat leaf rust (Nagarajan and Singh, 1990), sugarcane rust, wheat stem rust, and stripe rust (Wellings and Mcintosh, 2010) from the Indian subcontinent, resulting in the yield loss of global cereal crops (Narayanasamy, 1997). Other important pathogenic fungi, such as *Hemileia vastatrix*, cause coffee leaf rust (Bowden et al., 1971), and *Phakopsora pachyrhizi* causes soybean rust (Pan et al., 2006), which can spread from Africa and Asia to North America, respectively. Another typical biotrophic genus, *Microbotryum*, which is a smut fungus, showed transcriptional activity as well with rRNA:rDNA ratio equal to 3.91 in present study. The same as rust fungi, powdery mildew fungi and smut fungi are obligate parasites and mainly spread through the air; therefore, we believe that both of them remain transcriptional activity when spread in the air, which deserves high attention and more research in the future.

In addition, several putative plant pathogens have not been reported or distributed in China; for example, *Diatrype prominens* (average rRNA:rDNA ratio = 2.37), mainly distributed in North America according to the record of GBIF, is a host of *Arbutus menziesii* (Tedersoo et al., 2014). According to the previous study, *Ophiognomonia clavigignenti-juglandacearum* is mainly distributed in the United States and Canada, causing walnut canker, and are also parasitic to other species of *Juglans* (Nair et al., 1979). It was first found in Wisconsin and then spread to other states and Canada; was identified as an invasive species in North America in 1967; and may pose a serious threat to survival of white walnut. *Trechispora alnicola* can cause yellow ring disease on *Poa pratensis* (Wilkinson, 1987), distributed in over 24 countries or regions in Asia, Africa, South America, North America, and Oceania. *Uromycladium tepperianum* causes gall rust of *Acacia saligna*, which is native to Australia, and was used to prevent the expansion of *A. saligna* in South Africa (Wood and Morris, 2007). Currently, it is mainly distributed in Australia, South Africa, New Zealand, Indonesia, and New Caledonia based on the record on GBIF. Among the above pathogenic fungi, there were five species with potential transcriptional activity, i.e., *D. prominens* (average rRNA:rDNA ratio = 2.37) and *Septofusidium herbarum* (2.53) in Ascomycota, and *Pseudomicrostroma glucosiphilum* (1.55) and *U. tepperianum* (15.41), which suggest that they may be have spread over long distances in the air and may cause diseases in favorable conditions.

Altogether, our results indicated that survival in air is a common feature of plant pathogenic fungi; it covers all biotrophic, hemibiotrophic, and necrotrophic groups. While about 50% of the fungi were indicated in dormancy, the seasonal variation of the dormancy of the fungi needs to be investigated to understand whether a dormant group can last longer in the air or lacks a host.

Nevertheless, the limitation of compared rRNA/rDNA analysis must be considered, although it is useful for potential transcriptional activity research (Blazewicz et al., 2013). Due to differences in sample processing and/or insufficient sampling, deviation of rRNA:rDNA ratio calculation will result, and therefore, other unknown reasons will cause the appearance of “phantom taxa,” which only exist in rRNA sequencing subset but not observed in rDNA sequencing subset (Klein et al., 2016), or active populations as will be misclassified as dormant (Steven et al., 2017). Additionally, quantification and direct metabolic activity analysis of major pathogenic fungi should be conducted to carry out risk assessment in future research.

#### Potentially Transcriptionally Active Human Pathogens and Animal Pathogens in the Air

In our complex changing world, some environmental health problems are not straightforward to identify until a very serious health impact occurs (Lai et al., 2009). We identified human pathogenic and animal pathogenic fungi in the atmosphere in present study: four human pathogens, i.e., genera *Candida* and *Microascus*. *Candida* normally lives on the skin and inside the body and can cause infections if it grows out of control or if it enters deep into the body (Fotos and Hellstein, 1992). *Microascus* species have emerged as significant invasive pathogens in the immunocompromised patient. They are also frequently recovered as the *Scopulariopsis* anamorph in more superficial settings and as agents of onychomycosis. For example, the best-matched species is *Microascus cirrosus* (Per. Ident = 99.67% by BLAST in NCBI), which causes onychomycosis (De Vroey et al., 1992) and postoperative fungal infection (Krisher et al., 1995; Miossec et al., 2011). There are other two putative fungi: *Rhizomucor pusillus* (with Per. Ident = 99.3–100%, but highest Per. Ident with *Rhizomucor miehei* was only 96.52%) is a conditionally pathogenic fungus that causes inflammation and tissue degeneration and necrosis, which can spread directly in the organism and spread in blood (Ma et al., 1992); *Lichtheimia corymbifera* causes mucormycosis, which can spread widely to the brain, lungs, gastrointestinal tract, and heart (Lu et al., 2008; Garcia-Hermoso et al., 2009; Bellanger et al., 2010). Among which, rRNA:rDNA ratios of *M. cirrosus* and *L. corymbifera* were 4.56 and 4.36, respectively, which probably indicated their high risk of infection as they have high transcriptional activity, especially for *M. cirrosus*, which was active across the whole sampling season.

Similarly, the putative species *Acaromyces ingoldii* (Per. Ident = 99.04% by BLAST in NCBI, but Per. Ident of other matches were lower than 90%), as an animal pathogen, is currently distributed in Brazil, Panama, China, Israel, Japan, Philippines, Vietnam, and other countries or areas according to records on GBIF, with potential transcriptional activity (rRNA:rDNA ratio = 35.81) indicating the risk to some extent. As a result, we suggest that it is a necessity to strengthen the research on the potential active human/animal pathogens in the air and to improve the awareness of risk control before health hazards.

#### Other Fungi With Potential Transcriptional Activity

There were 36 putative species of Basidiomycota and five putative species of Ascomycota in macrofungi (mushroom/wood-rotting fungi). However, only seven of them were transcriptionally active, which was the lowest proportion of all categories, suggesting that spores of macro-fungi in air were dormant. Other potential active species of different functional categories, including lichenized fungi and saprophytic fungi, probably play broad and important roles on Earth such as affecting precipitation, carbon, and nitrogen cycles. While there are few studies focusing on them, so far, results in them having an important ecological function have not been discovered yet.

## Conclusion

In the present study, we revealed that there are considerable amount of active fungi *via* RNA-based approach and imaging cytometry simultaneously. RNA-based amplicon showed that Ascomycota dominated the active community, while Basidiomycota have low transcriptional activity in the active community. The structure of the active community of atmospheric fungi was significantly different from that of total community. For the first time, we deeply analyzed the potential transcriptional activity of airborne fungi from different ecological and functional categories and figured out that plant pathogenic fungi are the dominant group in the air, and more than 50% were potentially active. Our results further indicated that survival in air is a common feature of plant pathogenic fungi regardless of biotrophic, hemibiotrophic, and necrotrophic groups. Moreover, it is essential to strengthen the research on the potential active pathogens of humans and animals in the air to prevent risks before health hazards; also, rare species with potential transcriptional activity in the atmosphere should not be underestimated and/or ignored, which may play an important role in the biosphere, while little research has been done and their important ecological functions remain largely undiscovered. Considering the genetic and species diversity of pathogenic fungi in the air, resistant crop and varieties should be selected for distribution based on local and seasonal variations of pathogenic fungi dynamics across a broader geographic region in agricultural production. This study provides a new perspective for more comprehensive understanding of the atmospheric fungal community, as well as the scope of biodiversity and biogeography extension, and to help to establish awareness of microbial function and safety.

## Data Availability Statement

The amplicon sequencing reads have been deposited in the NCBI SAR with the accession number SUB9585712 and the Bioproject number PRJNA727682.

## Author Contributions

YC, YW, and LL conceived and designed the experiments. ZH, YZ, and LW contributed to the sample collection and species information query for annotation. YC, XZ, and JD carried out all the laboratory work. YC and XZ contributed in the data analysis and writing. CL contributed in the funding acquisition and supervision. SJ contributed to the writing—reviewing and editing. All authors contributed to the article and approved the submitted version.

## Conflict of Interest

The authors declare that the research was conducted in the absence of any commercial or financial relationships that could be construed as a potential conflict of interest.

## Publisher’s Note

All claims expressed in this article are solely those of the authors and do not necessarily represent those of their affiliated organizations, or those of the publisher, the editors and the reviewers. Any product that may be evaluated in this article, or claim that may be made by its manufacturer, is not guaranteed or endorsed by the publisher.

## Footnotes

1
http://www.funguild.org/

2
http://www.nmc.cn/

3
http://www.tianqihoubao.com/aqi/kunming-201507.html
