# Supplementary material for: RNA-Based Analysis Reveals High Diversity of Plant-Associated Active Fungi in the Atmosphere
Source: Front Microbiol. 2021 Aug 31;12:683266. doi: 10.3389/fmicb.2021.683266 (PMC8438332; doi:10.3389/fmicb.2021.683266)
Supplement: Supplementary file 1 [file Data_Sheet_1.docx]

Supplementary Material

RNA-based Analysis Reveals High Diversity of Plant Associated Active Fungi in the Atmosphere

Yan Chen^1†^, Xishen Zhu^1†^, Ziqiong Hou^1^, Yi Wang^1^, Yunying Zhou^1^, Ling Wang^1^, Lin Liu^1^, Jingrong Duan^1^, Sauban Musa Jibril^1^ and Chengyun Li^1*^

^1^State Key Laboratory for Conservation and Utilization of Bio-Resources in Yunnan, Yunnan Agricultural University, Kunming, China

^*^Correspondence: Chengyun Li, [licheng_yun@163.com](mailto:licheng_yun@163.com)

†These authors have contributed equally to this work and share first authorship

**Supplementary Table S1.** Air quality data released by the local government during the sampling period from January 1^st^ to July 31^st^ of 2018.

**Supplementary Table S2.** Barcode sequence used in each sampling for library preparation and sequencing.

| **Sample ID** | **Forward (primer)-Barcode** | **Reverse (primer)-Barcode** |
| --- | --- | --- |
| YN1801Fd | ATCACG | ACTGAT |
| YN1802Fd | CGATGT | ATGAGC |
| YN1803Fd | TTAGGC | ATTCCT |
| YN1804Fd | TGACCA | CAAAAG |
| YN1805Fd | ACAGTG | CAACTA |
| YN1806Fd | GCCAAT | CACCGG |
| YN1807Fd | CAGATC | CACGAT |
| YN1801Fc | ACTTGA | CACTCA |
| YN1802Fc | GATCAG | CAGGCG |
| YN1803Fc | TAGCTT | CATGGC |
| YN1804Fc | GGCTAC | CATTTT |
| YN1805Fc | CTTGTA | CCAACA |
| YN1806Fc | AGTCAA | CGGAAT |
| YN1807Fc | AGTTCC | CTAGCT |

Note: “YN1801Fd, YN1802Fd, YN1803Fd, YN1804Fd, YN1805Fd, YN1806Fd and YN1807Fd” represented samples that were amplicon sequenced via rDNA from January to July of 2018, respectively; YN1801Fc, YN1802Fc, YN1803Fc, YN1804Fc, YN1805Fc, YN1806Fc and YN1807Fc represented samples that were amplicon sequenced via RDNA from January to July of 2018, respectively.

**Supplementary Table S3. Data preprocessing statistics and quality control information**

| **Sample ID** | **Raw reads(#)** | **Clean Reads(#)** | **Base(nt)** | **AvgLen(nt)** | **Q20** | **GC%** | **Effective%** |
| --- | --- | --- | --- | --- | --- | --- | --- |
| YN1801Fd | 86897 | 81306 | 24959974 | 306 | 79 | 47.19 | 93.57 |
| YN1802Fd | 87989 | 80214 | 24619349 | 306 | 81.37 | 48.09 | 91.16 |
| YN1803Fd | 84111 | 80103 | 24583820 | 306 | 82.72 | 48.37 | 95.23 |
| YN1804Fd | 61824 | 57677 | 17880088 | 310 | 63.84 | 49.08 | 93.29 |
| YN1805Fd | 85480 | 80203 | 24664767 | 307 | 80.11 | 48.14 | 93.83 |
| YN1806Fd | 84901 | 80857 | 24786269 | 306 | 83.91 | 46.14 | 95.24 |
| YN1807Fd | 84952 | 80028 | 24550004 | 306 | 83.93 | 46.83 | 94.2 |
| YN1801Fc | 67088 | 63055 | 19392432 | 307 | 78.17 | 47.46 | 93.99 |
| YN1802Fc | 84641 | 79386 | 24433434 | 307 | 79.51 | 48.47 | 93.79 |
| YN1803Fc | 74035 | 70154 | 21557245 | 307 | 78.83 | 48.59 | 94.76 |
| YN1804Fc | 69879 | 67110 | 20793805 | 309 | 65.27 | 49.01 | 96.04 |
| YN1805Fc | 88440 | 82742 | 25418086 | 307 | 78.4 | 47.64 | 93.56 |
| YN1806Fc | 86780 | 79359 | 24344854 | 306 | 83.01 | 47.77 | 91.45 |
| YN1807Fc | 92241 | 90273 | 27492246 | 304 | 83.04 | 46.12 | 97.87 |

**Supplementary Table S4. T-test results of α-diversity indices between total fungi communities and potential active fungi communities in the air**

| **α-diversity indices** | **Total fungal community** | **Active fungal community** | **T** | **Sig.** |
| --- | --- | --- | --- | --- |
| **Richness** | 198.57±35.50 | 168.86±38.42 | 1.503 | 0.159 |
| **Shannon** | 5.29±0.75 | 5.26±0.27 | 0.105 | 0.918 |
| **Simpson** | 0.91±0.07 | 0.94±0.01 | -1.131 | 0.280 |
| **Chao1** | 250.6±60.63 | 228.36±60.65 | 0.686 | 0.506 |
| **ACE** | 274.09±63.81 | 252.98±69.90 | 0.590 | 0.566 |
| **Goods coverage** | 0.97±0.01 | 0.97±0.01 | -1.000 | 0.337 |
| **PD whole tree** | 11.87±1.63 | 10.11±1.74 | 1.951 | 0.075 |

Note: Data presented as the mean ± S.D.

**Supplementary Table S5.** Operational taxonomic unit (OTU) table including taxonomic classification and relative abundance of read

**Supplementary Table S6.** List of fungi species with potentially transcriptional activity


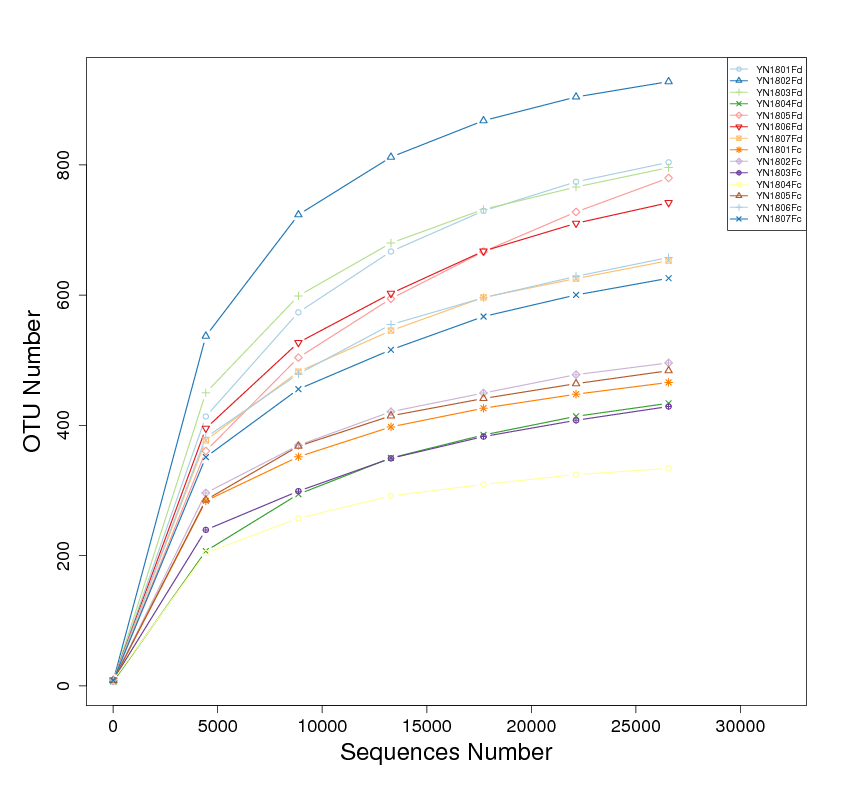


**Supplementary Figure S1.** Dilution curve showing the rationality of sequencing


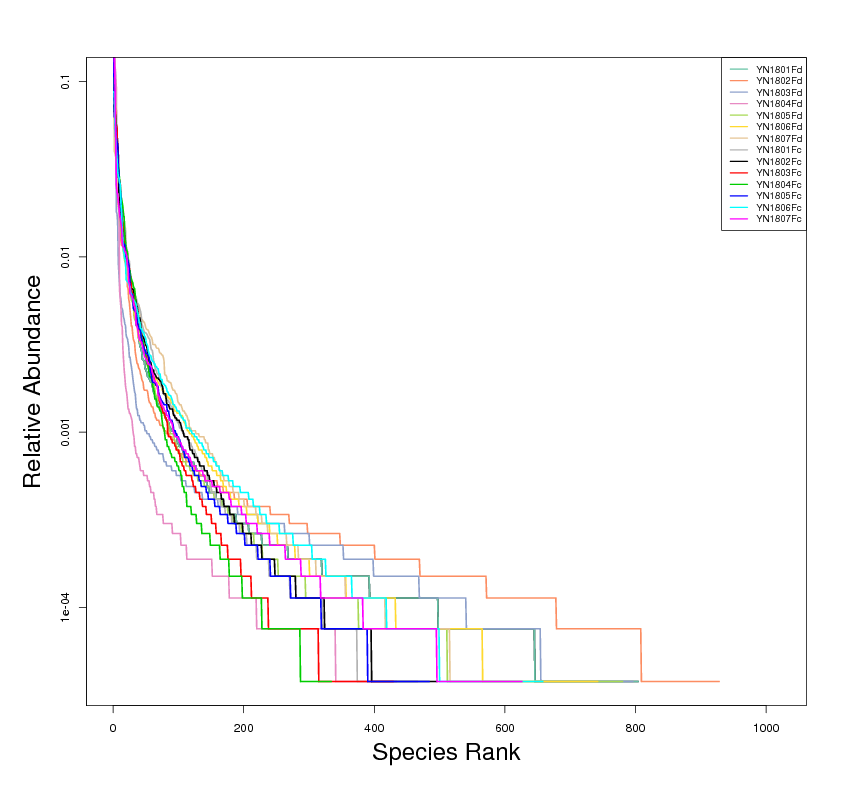


**Supplementary Figure S2.** Rank Abundance curve showing the species richness and evenness in the samples


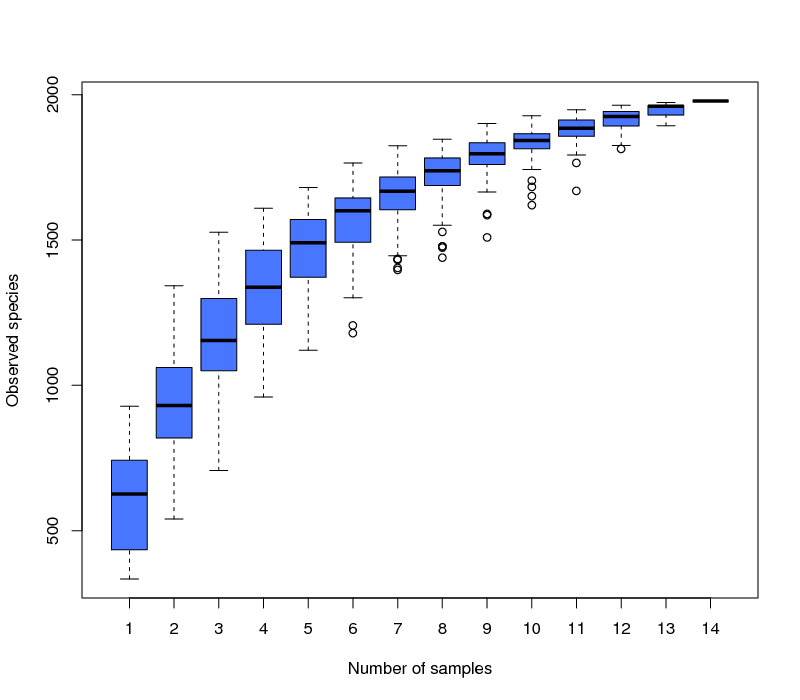


**Supplementary Figure S3.** Species accumulation curve showing the number of detected OTUs as a function of number of samples


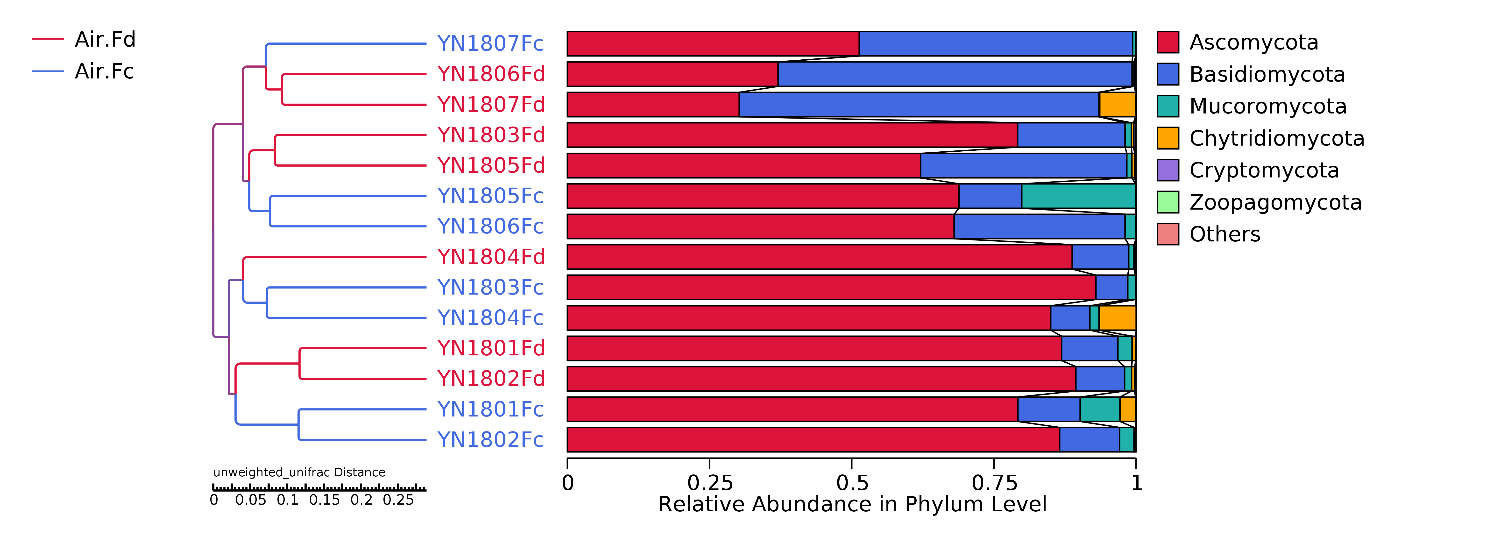


Supplementary Figure S4. Hierarchical clustering tree of total and active fungal community in the air based on UPGMA by unweighted unifrac distances at the phylum level


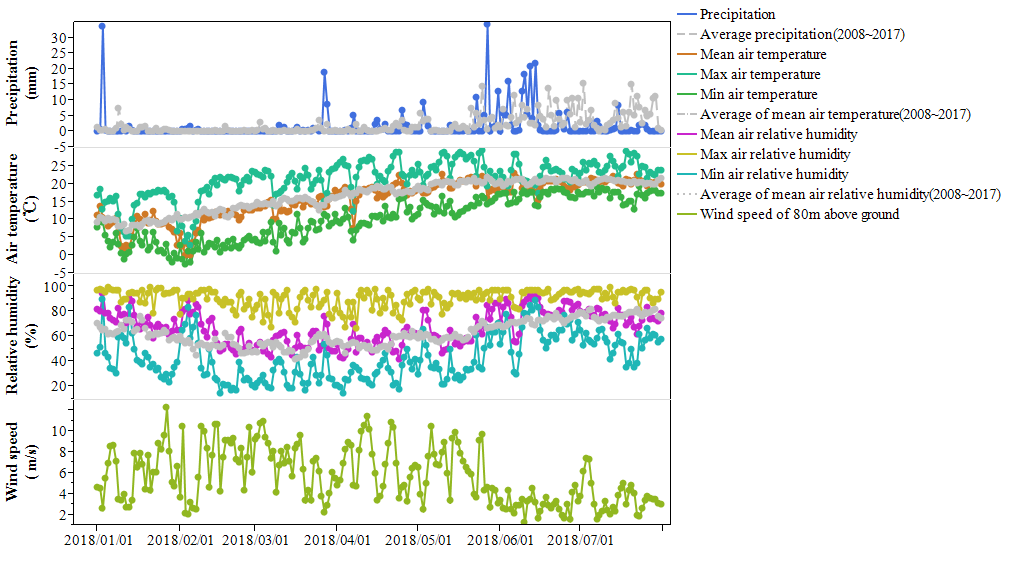


**Supplementary Figure S5.** Dynamics of precipitation, air temperature, air relative humidity and mean wind speed during January to July of 2018
